# Single-molecule mechanics of protein-labelled DNA handles

**DOI:** 10.3762/bjnano.7.16

**Published:** 2016-01-29

**Authors:** Vivek S Jadhav, Dorothea Brüggemann, Florian Wruck, Martin Hegner

**Affiliations:** 1 CRANN – The Naughton Institute, School of Physics, Trinity College Dublin, Dublin, Ireland; 2 Department of Physics, Northeastern University, Boston, MA, USA; 3 Institute for Biophysics, University of Bremen, Bremen, Germany

**Keywords:** DNA handles, optical tweezers, protein labels, single molecule

## Abstract

DNA handles are often used as spacers and linkers in single-molecule experiments to isolate and tether RNAs, proteins, enzymes and ribozymes, amongst other biomolecules, between surface-modified beads for nanomechanical investigations. Custom DNA handles with varying lengths and chemical end-modifications are readily and reliably synthesized en masse, enabling force spectroscopic measurements with well-defined and long-lasting mechanical characteristics under physiological conditions over a large range of applied forces. Although these chemically tagged DNA handles are widely used, their further individual modification with protein receptors is less common and would allow for additional flexibility in grabbing biomolecules for mechanical measurements. In-depth information on reliable protocols for the synthesis of these DNA–protein hybrids and on their mechanical characteristics under varying physiological conditions are lacking in literature. Here, optical tweezers are used to investigate different protein-labelled DNA handles in a microfluidic environment under different physiological conditions. Digoxigenin (DIG)-dsDNA-biotin handles of varying sizes (1000, 3034 and 4056 bp) were conjugated with streptavidin or neutravidin proteins. The DIG-modified ends of these hybrids were bound to surface-modified polystyrene (anti-DIG) beads. Using different physiological buffers, optical force measurements showed consistent mechanical characteristics with long dissociation times. These protein-modified DNA hybrids were also interconnected in situ with other tethered biotinylated DNA molecules. Electron-multiplying CCD (EMCCD) imaging control experiments revealed that quantum dot–streptavidin conjugates at the end of DNA handles remain freely accessible. The experiments presented here demonstrate that handles produced with our protein–DNA labelling procedure are excellent candidates for grasping single molecules exposing tags suitable for molecular recognition in time-critical molecular motor studies.

## Introduction

Most mechano-enzyme and protein–DNA interaction studies using optical tweezers (OT) are performed in a dumbbell configuration, where a single or double-stranded DNA molecule or protein is tethered between two optically trapped beads. Alternatively, one of the two beads can be held by a micropipette via suction in a single optical trap arrangement [1]. The DNA strand either interacts with a protein or mechano-enzyme directly or it serves as a spacer and a handle to isolate and tether a single mechano-enzyme of interest at a distance from the second bead. We here focus on the latter case and present a novel assay for reliably producing protein-labelled DNA hybrids (PDHs) to be used as molecular spacers and handles, exhibiting the necessary stability for time-critical displacement and force measurements. Streptavidin and neutravidin were used for protein labelling, acting as a molecular connection between the DNA spacer and the experimental target.

When a certain protein or enzyme of interest is tethered to surface-modified beads it is desirable to avoid unspecific surface interactions between the optically trapped spheres. This can be achieved by using single molecular handles. The use of DNA handles as molecular spacers is well documented in literature [2–8]. It may also be necessary to keep the two beads at a certain distance from each other during measurements involving large displacements, especially in dual-trap optical tweezers prone to polarization scrambling induced crosstalk [9]. In a stable and well-aligned instrument, the magnitude of the parasitic signal due to optical crosstalk is greatest when both traps overlap and reduces rapidly with increasing trap separation, oscillating about some mean value. By designing DIG-dsDNA-biotin handles (DHs) of specific lengths, one can choose a certain trap separation during experiments that minimizes contributions due to crosstalk.

DH lengths of 1000, 3034 and 4056 bp were chosen for the PDHs in this study. Short handles with greater stiffness could be produced quite easily and increase the signal-to-noise ratio (SNR) in high-resolution measurements [10]. We previously presented optical force rupture measurements of the nascent polypeptide chain from biotinylated ribosomes, which were specifically bound to surface-modified polystyrene beads [11]. While the ribosome was translating, the biotinylated lysine residue at the beginning of the nascent polypeptide chain was labelled in situ with streptavidin. This arrangement enabled us to grasp the protein chain with a DIG-DNA-Bio handle of 4056 bp length. Although we were able to study the rupture forces using this approach, the labelling of the nascent proteins during translation was not very efficient. To overcome these problems the development of mechanically stable DHs with pre-labelled protein ends were required. The constructs should enable efficient single hook-ups with the biotinylated tag of the probed biomolecule (e.g., nascent protein chain). Here, we present a labelling technique of DIG-DNA-Bio with two different proteins, streptavidin and neutravidin. Using optical tweezers the mechanical characteristics and the dissociation times of the novel DNA–protein hybrids were studied in various physiological buffers.

## Experimental

### Protein labelling of dsDNA and coupling to polystyrene beads

PCR amplification of plasmid pTYB1 (7477 bp, New England Biolabs, Ireland (NEB)) with 5’-biotin (5’-AAT TCT TGA AGA CGA AAG GGC GGC-3’ for 4056 bp DNA and 5’-GGA TAC GAC GAT ACC GAA GAC AGC-3’ for 3034 bp DNA) and 5’-thiol or 5’-DIG end-modified primers (both have the sequence 5’-TGT AAC TCG CCT TGA TCG TTG GGA-3’) were used to prepare DNA molecules (4056 and 3034 bp). BSAI linearized pNEB193 plasmids (2713 bp, NEB) were used to produce the 1000 bp long dsDNA handles. Here the primers 5’-biotin CAA CTG TTG GGA AGG GCG ATC-3’ and 5’-DIG-CTG TTA CCA GTG GCT GCT GCC-3’ were used. The Q5-DNA polymerase (NEB) was used throughout the experiments with appropriate annealing temperatures and elongation times to produce the dsDNA fragments in a high purity and quantity. The DIG-DNA-Bio (ca. 100 nM) strands were incubated with either streptavidin (Sigma-Aldrich, Arklow, Ireland) or neutravidin (Pierce, Fisher Scientific, Dublin, Ireland) (both with approx. 60 kDa = 9.96 × 10^−20^ g each) at different ratios ranging from 100 to 500 proteins per DNA molecule. During incubation (30 min up to 48 h) the reactions were gently shaken to ensure efficient protein binding at the biotin end groups of the DHs at room temperature. Unbound proteins were removed using a ChargeSwitch^®^ cleaning kit (Invitrogen, Bio-Sciences, Dun Laoghaire, Ireland). For an enhanced yield we modified the cleaning kit protocol and increased the incubation time at the binding and elution step to 15 min. The successful labelling of the DHs with proteins, compared with unlabelled DHs, was confirmed via electrophoresis in a 1% (SeaKem^®^ Gold, Lonza Rockland, USA) and 1.8% agarose gel (NuSieve™ GTG™ Agarose, Lonza Rockland, USA) with 1× Tris-acetate-EDTA buffer (Eppendorf AG, Hamburg, Germany) using a 1 kb DNA ladder (NEB & Promega). We utilized UV–vis spectroscopy (Nanodrop spectrophotometer ND-1000, Fisher Scientific, Dublin, Ireland) to measure the concentration of the DHs and PDH constructs. These measurements were also indicative for the presence of protein after 90 min of incubation and the quality of the subsequent cleaning using the ChargeSwitch^®^ kit.

**Figure 1:**
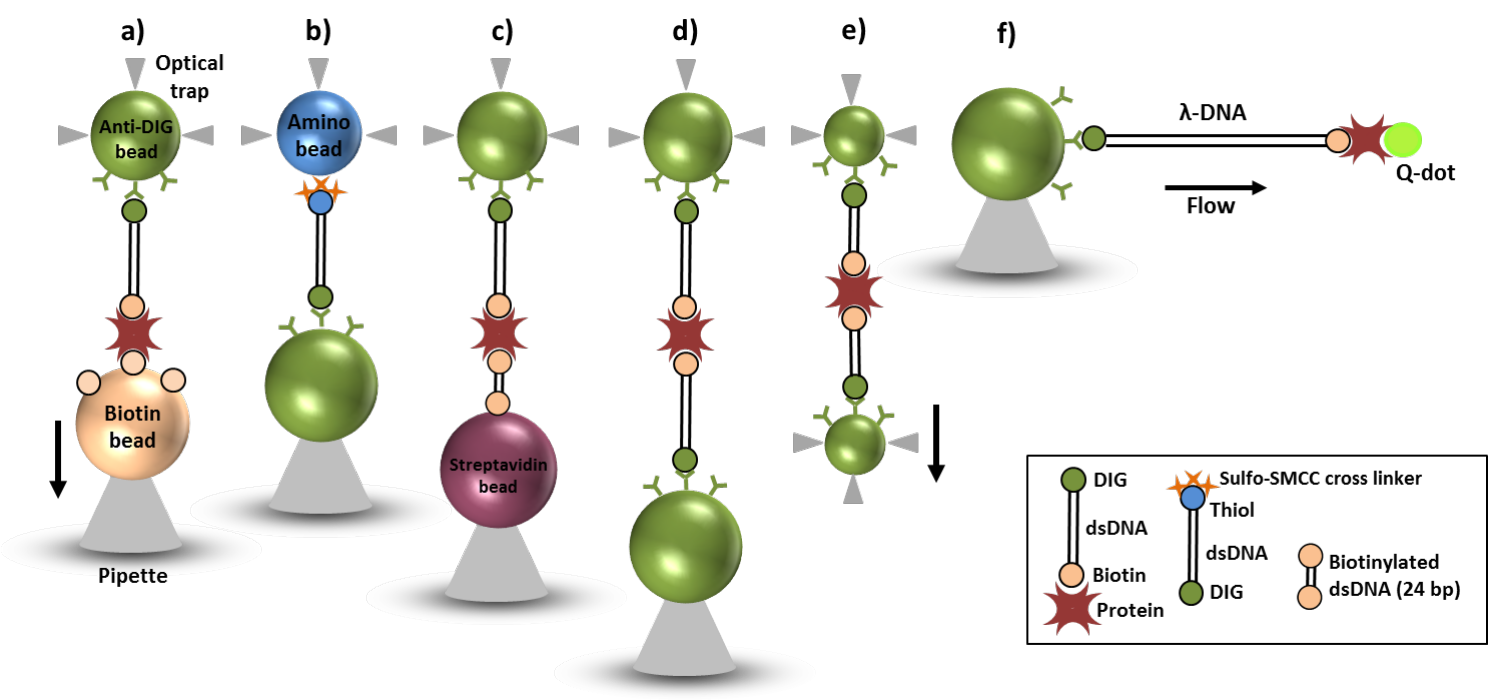
Bead arrangements in different optical force measurements. a) PDHs tethered to an anti-DIG bead (2.9 μm diameter) in the optical trap pulled versus a biotinylated polystyrene bead (3.2 μm). b) DIG-DNA-Thiol covalently bound to an amino bead (2.1 μm) in the trap pulled versus an anti-DIG bead (3.4 μm) in the pipette. c) Handle experiment with long DNA (3034 bp or 4056 bp) molecules: The pipette holds a streptavidin bead (3.1 μm) modified with short dsDNA linkers (24 bp) and is probed with PDHs. d) Double handle experiment with 4056 bp or 3034 bp DHs coupled to anti-DIG bead (3.4 μm) in the pipette and an anti-DIG bead (2.9 μm) of different diameter with PDHs tethered to it is optically trapped. The biotin end and the protein label are interconnected in situ to form a double-length DNA handle. e) Double-handle experiment using sub-micron beads (0.84 μm each) and short DNA molecules (1000 bp) utilizing a high-resolution dual trap OT. f) End-labelled DIG-λ-DNA-biotin bound to an anti-DIG bead (3.4 μm) held by a micropipette. Flow forces elongate the λ-DNA molecule that is tethered at one end to the microsphere. The streptavidin-modified end (labelled with quantum dots) is freely floating in the microfluidic chamber.

To anchor the DIG end of the DHs we have surface-modified polystyrene beads with the antibody anti-DIG. Protein G beads of 2.9 µm or 840 nm diameter (Spherotech, Lake Forest, IL, USA) were washed by pelleting three times in PBS buffer. Anti-digoxigenin antibodies (200 µg/mL in PBS; Roche Diagnostics, Rotkreuz, Switzerland) were incubated for 1 h at room temperature and washed once by pelleting in 0.05 M sodium tetraborate, pH 8.2. The anti-DIG antibodies were then cross-linked to the protein G beads using 10 mg/mL DMP crosslinker (Pierce/Fisher Scientific, Dublin, Ireland) in 0.2 M triethanolamine (Sigma, Arklow, Ireland). The DMP cross-linking reaction was then quenched in 0.1 M ethanolamine at pH 8.2 for 5 min pelleted and resupended in 0.05 M sodium tetraborate, pH 8.2. Non-crosslinked anti-DIG antibodies were removed by pelleting twice in 0.1 M glycine, pH 2.8. Finally the anti-DIG can be stored at 4 °C in PBS containing 0.05% sodium azide and 0.1% tween 20. Alternatively, aldehyde/sulfate latex beads with 2 µm diameter (Invitrogen, Dublin, Ireland) were used, which had been covered with anti-DIG via passive adsorption. The coupling of PDHs to anti-DIG spheres was carried out at room temperature for 90 min with different coupling ratios as discussed in the result section. We used this standardised protocol in the entire study and were able to reproduce the result multiple times (more than about 250 times) with specific interactions using the OT setup. For DNA with DIG at the 5’ end and thiol at the 3’ end a PCR using the 7477 bp long plasmid pTYB1 was carried out with 5’-DIG end-modified and 5’-thiol end-modified primers. Subsequently, the thiol-end was covalently coupled to amino-beads with 2.1 µm diameter (Spherotech, Lake Forest, IL, USA) using a sulfo-SMCC cross linker (Fisher Scientific, Dublin, Ireland). DIG-DNA-Thiol was activated for 1 h at room temperature with a ratio of approx. 1000 SMCC molecules per DNA as previously described [8]. Excess of SMCC was removed using a ChargeSwitch^®^ cleaning kit, and the activated DNA was coupled to amino beads with an average of 50 DNA molecules per bead (Figure 1b).

A dsDNA linker of 24 bp length with biotinylated 3’- and 5’-ends was formed from two complementary single DNA strands (sequence: biotin-5’-AAT TCT TGA AGA CGA AAG GGC GGC-3’-biotin and its complementary sequence 5’-GCC GCC CTT TCG TCT TCA AGA ATT-3’, Microsynth, Balgach, Switzerland) via thermal annealing. This linker was added to streptavidin beads of 3.1 µm diameter at a concentration of 1 μM to cover the bead surface with biotinylated linkers. In subsequent optical force measurements these bio-linker-streptavidin beads were used to enable specific interactions with the protein end of our PDHs (Figure 1c).

Lambda-dsDNA 48502 bp (Roche, Lifescience, Switzerland) was modified on either end at its cos-sites by ligating two oligo-nucleotides (5’-GGG CGG CGA CCT-3’-Bio and DIG-3’-CCC GCC GCT GGA-5’) in an overnight reaction at 4 °C with T4 DNA ligase (Roche, Lifescience, Switzerland). Excess oligonucleotides were cleaned off by ethanol precipitation using sodium acetate as counter ions. Modified λ-DNA was resuspended in 10 mM HEPES, 1 mM EDTA buffer, pH 7.2. The biotinylated end was modified with a 100× concentration of Qdot 525 streptavidin conjugate (Life technologies, Dublin, Ireland) (Figure 1f). Excess Qdots were washed out during the OT experiment.

### Physiological buffers

Force measurements were performed in various protein translation buffers [2,11–12] in a microfluidic cell. All chemicals used in the buffer preparation were of a high purity grade and were used as received without further purification. Sodium chloride (≥98%), HEPES (BioUltra ≥99.5%), magnesium acetate tetrahydrate (99%), ammonium acetate (98%), 2-mercaptoethanol (BioUltra ≥99.0%) and DL-dithiothreitol (BioUltra ≥99.5%) were provided from Sigma-Aldrich (Dublin, Ireland). All aqueous solutions were prepared using nanopure water from a Millipore Milli-Q system, additionally filtered with 0.22 µm pore size filters. We used TICO buffer (20 mM HEPES-KOH, pH 7.5, 6 mM magnesium acetate, 30 mM ammonium acetate, 4 mM 2-mercaptoethanol) to examine various coupling ratios of PDHs to anti-DIG beads for the subsequent biotin-streptavidin binding in OT experiments. All experiments were also performed in TICO buffer with an increased Mg content of 12 mM (high Mg TICO). Control experiments were carried out in standard buffer (150 mM NaCl, 10 mM HEPES, pH 7.5) and in DTT buffer (40 mM HEPES-KOH, pH 7.5, 60 mM NH_4_Cl, 10 mM magnesium acetate, 1 mM DTT and 3.6 mM 2-mercaptoethanol) (adapted from [12]).

### Optical force measurements

Measurements of the DNA mechanics were performed with a previously described counter-propagating dual-beam OT setup [13] (Figure 1a–d, f) and a high resolution dual trap OT device [14] (Figure 1e). The PDHs coupled to anti-DIG spheres were injected into a microfluidic cell chamber placed in-between two identical water immersion microscope objectives and the beads were optically trapped. For experiments with PDHs (to confirm protein modification (streptavidin or neutravidin) at the biotin end of the DNA handle) a biotin coated bead of 3.2 µm diameter (Spherotech, Lake Forest, USA) was held in a micropipette by suction (Figure 1a).

An anti-DIG bead covered with the PDH constructs was injected and optically trapped. The protein label at the 3’ end of the bead-coupled DNA strand was tethered to a biotin-coated bead, by bringing both beads within close proximity of each other (a few hundred nanometres). The position of this pipette was controlled with a closed-loop piezoelectric element, during force–displacement measurements the bead in the pipette was moved away from the trapped bead at a constant velocity of 100 nm/s and force signals were low-pass filtered at 159 Hz. The setup directly measured the change in light momentum flux when the trapped bead experienced a lateral force [13]. Control experiments were carried out to check for specific interactions between the PDH constructs (bound to anti-DIG beads) and streptavidin surface-modified beads (3.1 µm diameter, Spherotech, Lake Forest, USA), no interactions were observed. Protein labelling at the biotinylated ends of the DNA constructs was essential, as ends that are not protein-labelled could lead to false positive interactions between the bare biotinylated DNA ends and streptavidin or neutravidin surface-modified beads or molecules.

To characterize the binding strength of the DIG::anti-DIG bond (the weakest link in the molecular construct) we trapped amino beads covalently coupled to DIG-DNA-Thiol handles and tethered those in situ to anti-DIG beads (2.9 µm diameter) held in place through suction by a pipette (Figure 1b). In the configuration shown in Figure 1c we could test the protein-modified end of the PDHs when no short dsDNA linker molecules were implemented (not shown) and test configurations in which a prospective biomolecular construct was directly grafted to the sphere surfaces. To simulate in situ tethering to complex biomolecules, we used PDHs to grasp single DNA molecules in situ (Figure 1d,e). For experimental configurations illustrated in Figure 1d, we held DHs tethered to anti-DIG beads (3.4 µm diameter) in the pipette. Then, smaller anti-DIG beads (2.9 µm diameter) with PDHs were optically trapped to obtain hook-ups between the protein label and the biotin end of the molecule at the pipette-held bead. In Figure 1e the same configuration was tested using the 1000 base pair PDHs in a dual-trap configuration. Because the in situ tethering took place between individual molecules suspended within the inter-bead liquid (Figure 1d,e) compared to the configurations shown in Figure 1a–c the hook-up times were generally longer, on the order of approx. 2 min.

For proof-of-concept that the PDH end remains freely accessible in liquids, the biotin end of the DH was labelled with streptavidin–Qdot conjugates. Then, they were anchored to anti-DIG beads (Figure 1f), and the position of individual Qdots under varying fluid flow velocities was monitored using an EMCCD camera (Andor Technology, Belfast, NI). Laminar fluid flow inside the microfluidic chamber constrained the free Qdot-modified end of the DNA within the image plane of the EMCCD camera (Figure 8). The distance between the bead surface and the position of the Qdot represented the fluid-flow dependent DNA extension, corresponding to the hydrodynamic drag experienced by the DNA molecule. The flow force induced stretching as presented here is consistent with the original experimental findings by Perkins et al. [15] and follows the theoretical description of Stigter et al. [16].

## Results and Discussion

### Protein labelling of dsDNA

**Figure 2:**
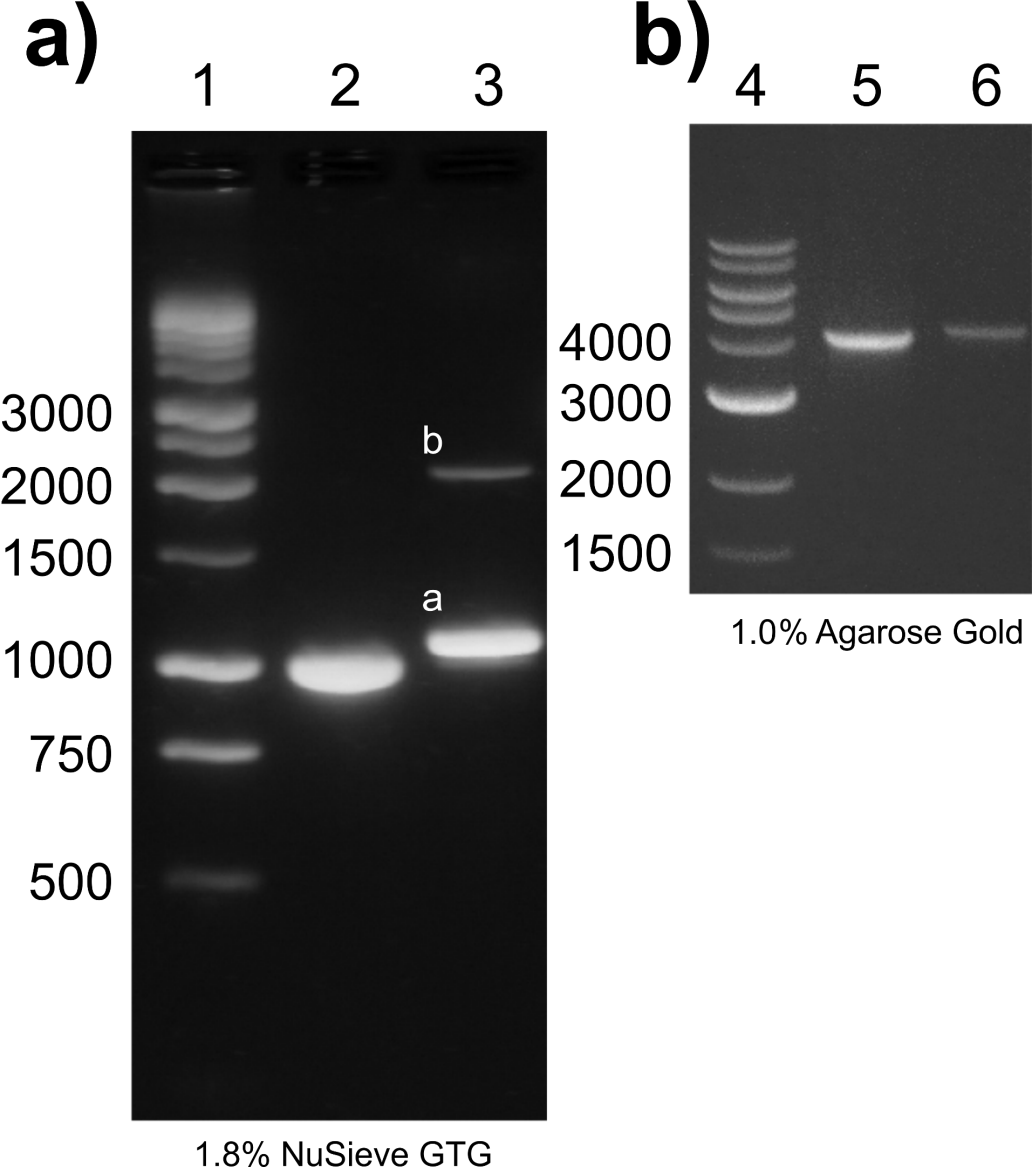
Quantitative analysis of labelled and unlabelled DNA. Gel electrophoresis [(1.8% a) (lane 1, 2 and 3) and 1% b) (Lane 4, 5 and 6)]: DHs of 1000 bp (lanes 2) and 4056 bp (lane 5) respectively, versus PDHs (lanes 3, 6) and a 1 kbp ladder [lane 1 (Promega) and lane 4 (NEB)]. In lane 3 some double-length dsDNA (2000 bp) are visible, linked through streptavidin (≈5% of the total PDHs) (compare to Figure 1d,e).

Labelling of protein to DIG-DNA-Bio (4056, 3034 and 1000 bp) was carried out as discussed in the Experimental section. The labelled and unlabelled PDHs were analysed using electrophoresis in a 1.8% (for 1000 bp) and 1% (for 4056 bp) agarose gel and UV–vis absorption spectra. The mobility of the PDHs was expected to be less than that of the unlabelled DIG-biotin strands and was confirmed with band shifts in agarose gel electrophoresis tests (Figure 2). 1.8% (a) and 1% gel (b) images are shown next to each other, these shifts are more prominent in gel lanes 2 and 3 where the bare 1000 bp dsDNA population is compared with streptavidin protein conjugated 1000 bp dsDNA. The proportional band shifts in the DNA–protein hybrid constructs with 4056 bp DHs are also seen in lane 5 and 6, where bare DIG-DNA-Bio is compared with protein-labelled DIG-DNA-Bio. This is within the limitation of the size/charge discrimination of the agarose gel technology. The PDHs analysed in lane 3 showed that a small portion of the modified dsDNA molecules formed double handles linked by streptavidin when an incubation ratio of 100:1 proteins per dsDNA was used. Densitometry analysis of the 1.8% gel image using ImageJ revealed that less than 5% of the molecules resulted in double length molecules. The integrated values of the analysed individual bands are: lane 2 – 12500 a.u., lane 3a – 11100 a.u. and 3b – 1100 a.u. Here, 1100 a.u. in lane 3b correspond to the double-length molecule of 2000 bp, consequently resulting in twice the fluorescence intensity. The densitometry analysis also confirmed that the purified PDH constructs were representing over 99% PDHs, and no observable ‘naked’ modified dsDNA remained in the sample.

The yield of PDHs after 90 min of incubation was approx. 10 nM when 100 nM of DHs were used, no significant increase in the yield of protein labelled DNA molecules was observed, when the reaction time was extended from 90 min to 48 h. Shorter incubations resulted in lower yields of PDHs. Reactions with nominal ratios of 300 and 500 protein molecules per DNA showed higher purification losses and yielded lower concentrations of PDHs than incubations where 100 proteins per DNA were used. Low coupling ratios such as 10, 20 and 50 proteins per DNA, resulted in very low specific hook-up rates to biotin beads/handles, probably due to the greater number of DNA molecules (>1) per protein. Also, the number of unlabelled DNAs could be too high, resulting in too many unwanted interactions with streptavidin-coated beads. We assume that the cleaning procedure with the ChargeSwitch^®^ kit becomes less efficient when coupling ratios greater than 100:1 are used in the reaction volume, since they lead to a higher total loss of PDHs during cleaning. These observations were independent of the respective protein (streptavidin or neutravidin) used.

### Optical force measurements with protein-labelled DNA

The overall rate of specific interactions was examined by modifying the coupling ratio of PDH to anti-DIG beads. During 90 min of incubation the various PDH constructs were tethered to anti-DIG beads (2.9 µm diam.) with nominal ratios of 20, 40 and 80 molecules per bead. Optical force measurements of these constructs were carried out in different buffers (Table 1) versus biotin microspheres (Figure 1a), and interaction rates as well as rupture and plateau forces were compared. The interaction rate was calculated by dividing the number of specific interactions (i.e., DNA hookup and elongation) and the number of approaches.

**Figure 3:**
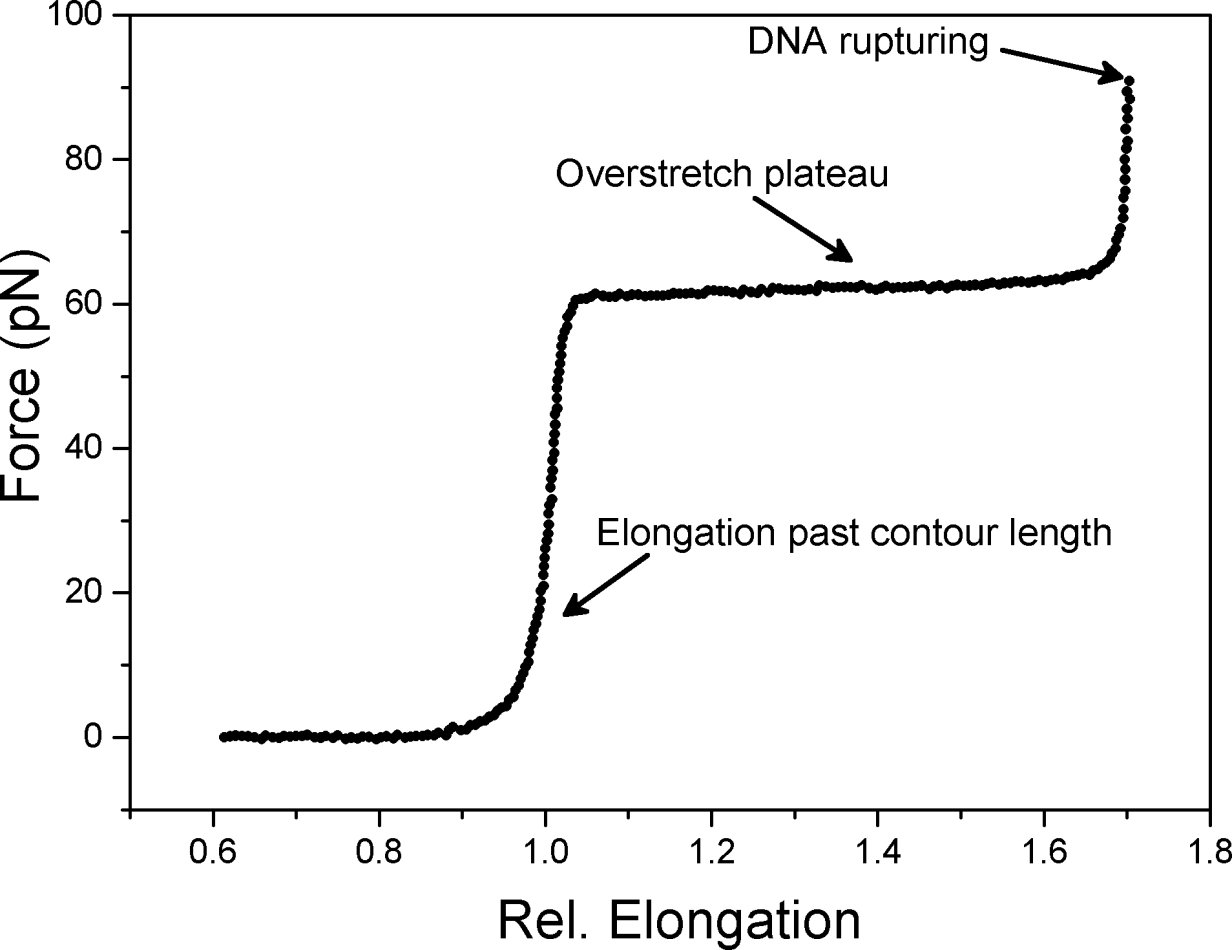
Characteristic force–extension curve. The biotin bead interacting with the protein moiety by molecular recognition is moved away from the DNA-labelled bead in the trap at a constant velocity of 100 nm/s until rupturing occurs. Lower pulling speeds, e.g., 10 nm/s and 50 nm/s were tested and resulted in similar force versus extension plots.

The force measurements repeatedly yielded evidence of specific protein–biotin interactions, often with typical force–extension curves (Figure 3). The PDHs were overstretched at around 60–65 pN, a force range in good accordance with previous studies [17–18]. In many experiments rupture forces below 60 pN were observed due to the increasing loading-rate before the dsDNA strands were overstretched. The weakest linkage of the molecular construct is between DIG and the anti-DIG antibody [19]. In TICO buffer we found a stretch modulus of ca. 800 pN while measurements in high Mg TICO yielded approx. 1000 pN an increase consistent with previous studies [17,20]. The persistence lengths of force curves measured in TICO and high Mg TICO are 50 ± 3.5 nm (SD) [18,21].

With the lowest coupling ratio of 20 DNA molecules tethered to one anti-DIG sphere (20:1) the successful hook-up rate was between 18% for neutravidin-DNA and 25% for streptavidin-DNA in TICO buffer. With high Mg TICO buffer both PDHs yielded 35% of specific DNA interaction. These interaction ratios were rather low for studying the force characteristics of PDHs. Subsequently, with a 80:1 coupling ratio, the number of specific force interactions increased from 25 to 50% for streptavidin and 18 to 64% for neutravidin in TICO buffer and 35 to 87% and 35 to 60% in high Mg TICO for streptavidin and neutravidin-DNA, respectively.

However, multiple rupture events were observed with this coupling ratio (80:1) because several DNA strands were tethered between the two beads. These multiple interactions impeded the study of single molecule mechanics. Ideal experimental conditions with protein–DNA handles allowing for single hook-up experiments were then found with 40:1 handle-to-bead coupling ratio (bead diameter 2.9 µm). For smaller beads (0.84 µm diam.) the optimal ratio of PDHs coupling to beads was determined to be around 100:1.

In TICO streptavidin–DNA yielded specific interactions in 52% of the measurements while neutravidin-DNA showed 49% interactions with the ratio 40:1. The interaction rates were greater in high Mg TICO (streptavidin-DNA 66%, neutravidin-DNA 63%) as compared to 6 mM TICO buffer. However, a strong tendency of unfavourable bead clustering was witnessed in the microfluidic channel of our flow cell when DNA tethering and optical trapping were performed in high Mg TICO buffer. For future experiments involving the ribosomal machinery we therefore recommend the use of TICO buffer.

For the optimum DNA tethering ratio of 40:1, we also performed optical force measurements in standard buffer and in DTT buffer (Table 1). In standard buffer very high interaction ratios were obtained (streptavidin-DNA 79%, neutravidin-DNA 81%), and clustering only occurred in 16–17% of the experiments for both protein labels. With DTT buffer the interaction rate was only 53% for neutravidin-DNA and 61% for streptavidin-DNA, and bead clusters were observed in 20–35% of the experiments. A nominal ratio of 40:1 enabled efficient single molecule force measurements (90%) with the complete protein-labelled DNA construct against biotinylated beads (Figure 1a).

**Table 1:** Interaction rate of streptavidin- and neutravidin-DNA coupled to anti-DIG beads with the ratio 40:1 in various buffers.

interaction ratio 40:1	TICO percentage % (absolute #)	high Mg TICO	standard buffer	DTT buffer

streptavidin	52% (48)	66% (52)	79% (97)	61% (89)
neutravidin	49% (70)	63% (67)	81% (68)	53% (72)

**Figure 4:**
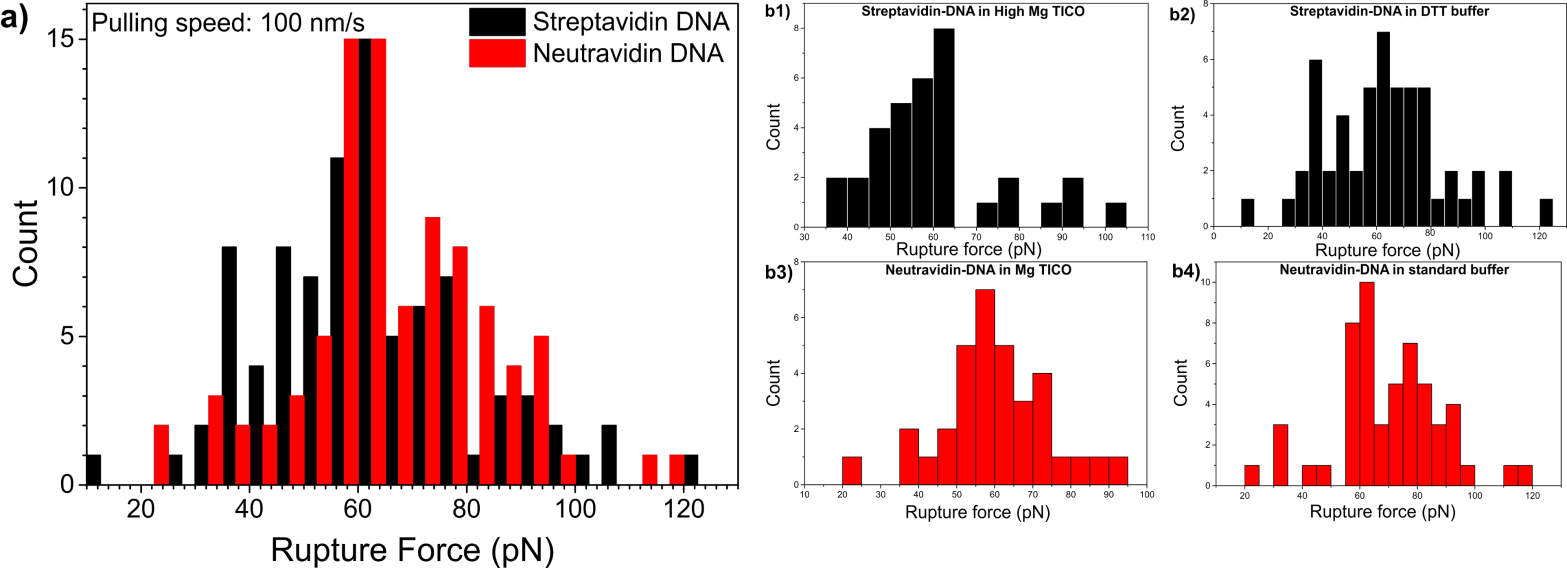
Selected force distributions for protein–DNA coupled to anti-DIG beads with the ratio 40:1. a) Combined rupture forces of streptavidin-DNA (b1 and b2) and neutravidin-DNA (b3 and b4) (*N* = 176; 88 measurements per PDH, bin size 5). b1) Rupture forces of streptavidin-DNA in high Mg TICO (34 force measurements, bin size 5). b2) Distribution of rupture forces for streptavidin–DNA in DTT buffer (54 measurements, bin size 5). b3) Rupture forces of neutravidin–DNA in TICO (34 measurements, bin size 5). b4) Rupture forces of neutravidin–DNA in standard buffer (54 measurements, bin size 5).

Rupture forces were analysed in all buffers for DHs with streptavidin or neutravidin labels, respectively. Rupture force distributions for both protein labels are shown in Figure 4. The rupture force distributions for both protein–DNA constructs were very wide with average rupture forces of around 60–65 pN in all buffers. Data representing the four individual buffer experiments could be merged since the individual histograms exhibited comparable appearance. It can be seen from the distribution peaks in Figure 4 that the rupture forces of streptavidin constructs were comparable to neutravidin hybrids. The pulling speed for all these measurements was constant at 100 nm/s but the loading rate varied depending on the relative elongation of the dsDNA at the rupturing point. The loading rate is the most relevant factor. Therefore in the molecular unbinding experiments shown here (Figure 4b1–b4) the rupture force distributions exhibited a maximum shortly at the highest loading rate before the force induced overstretch transition of the dsDNA at about 62 pN [17,22]. Maximum rupture forces of up to 120 pN occurred when the protein-labelled molecules could be stretched past the overstretch plateau. Considering the weaker nature of the non-covalent DIG::anti-DIG bond compared to covalent coupling where up to 200 pN can be reached [23–24], rupture forces of 120 pN underlines the mechanical strength of the protein-labelled molecules designed in this study.

**Figure 5:**
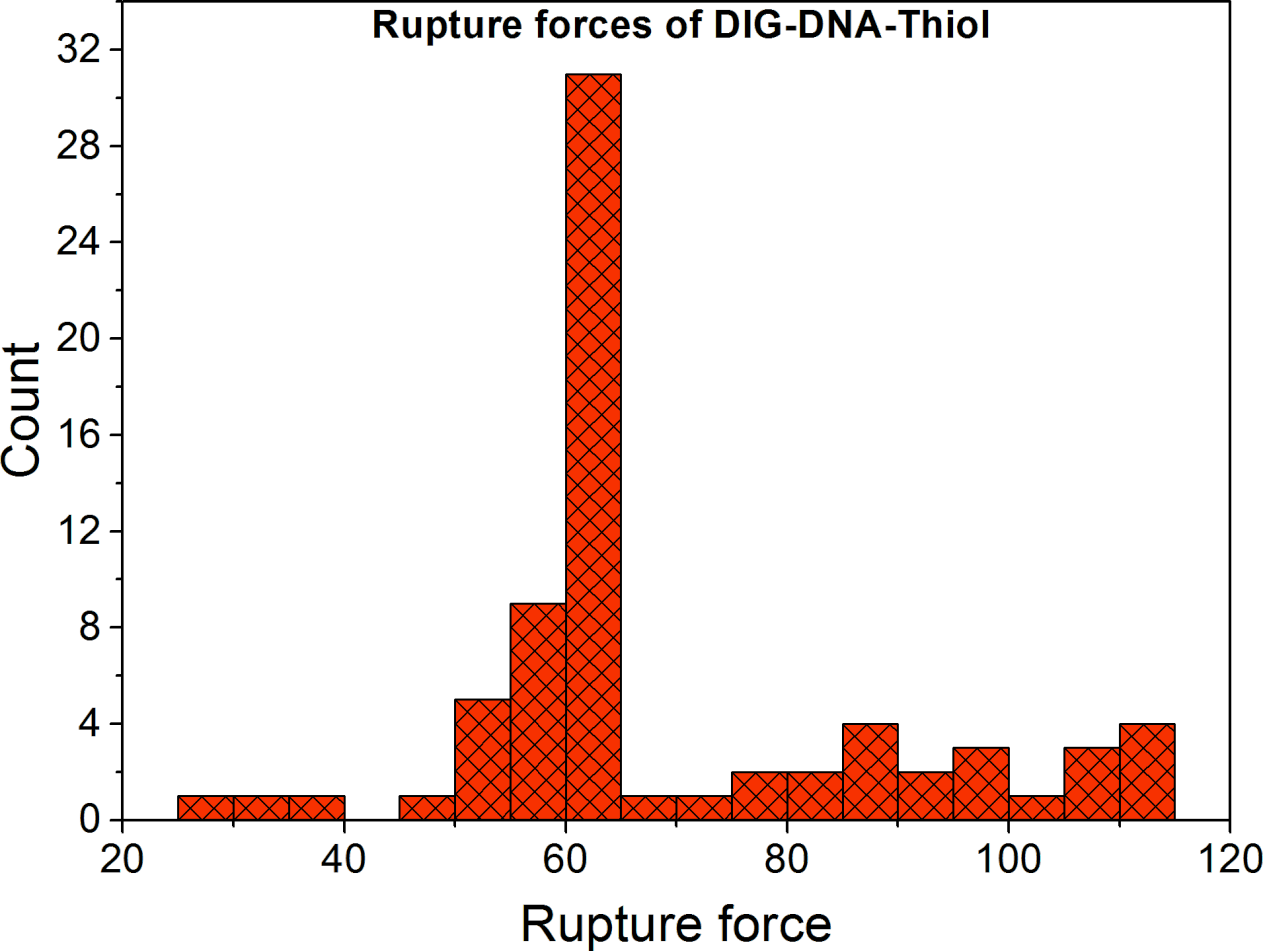
Optical force measurements of DIG-DNA-Thiol. Distribution of rupture forces for DIG-DNA-Thiol pulled versus anti-DIG beads (72 measurements, bin size 5). The average rupture force is around 60 pN with maximum forces reaching up to 115 pN. Pulling speed 100 nm/s.

To characterise the strength of the non-covalent DIG::anti-DIG coupling we studied the force characteristics of DIG-DNA-Thiol handles that were covalently coupled to amino beads with an average ratio of 50 DNA molecules per amino bead [8]. The construct was expected to rupture at the DIG::anti-DIG bond because the covalent thiol-amino bond is the more durable link in this configuration [23,25]. We pulled the DIG-Thiol handles versus anti-DIG beads (Figure 1b) in TICO and in standard buffer. The distribution of rupture forces for the DIG-Thiol constructs is shown in Figure 5. Most ruptures occurred at around 60 pN with maximum rupture forces of up to 115 pN. This force distribution accords with the results obtained using our PDHs, thus indicating that the DIG::anti-DIG bond is the weakest link in these constructs and the place where the molecules rupture [24]. When the anti-DIG antibody was not covalently linked to the protein G spheres, the average rupture forces were also observed to be around 60 pN. This observation indicates that the protein G interacting with the Fc part of the antibody ruptures earlier when no DMP crosslinking step was performed. Therefore, covalent crosslinking of the antibody to beads that are modified with protein G is advised.

**Figure 6:**
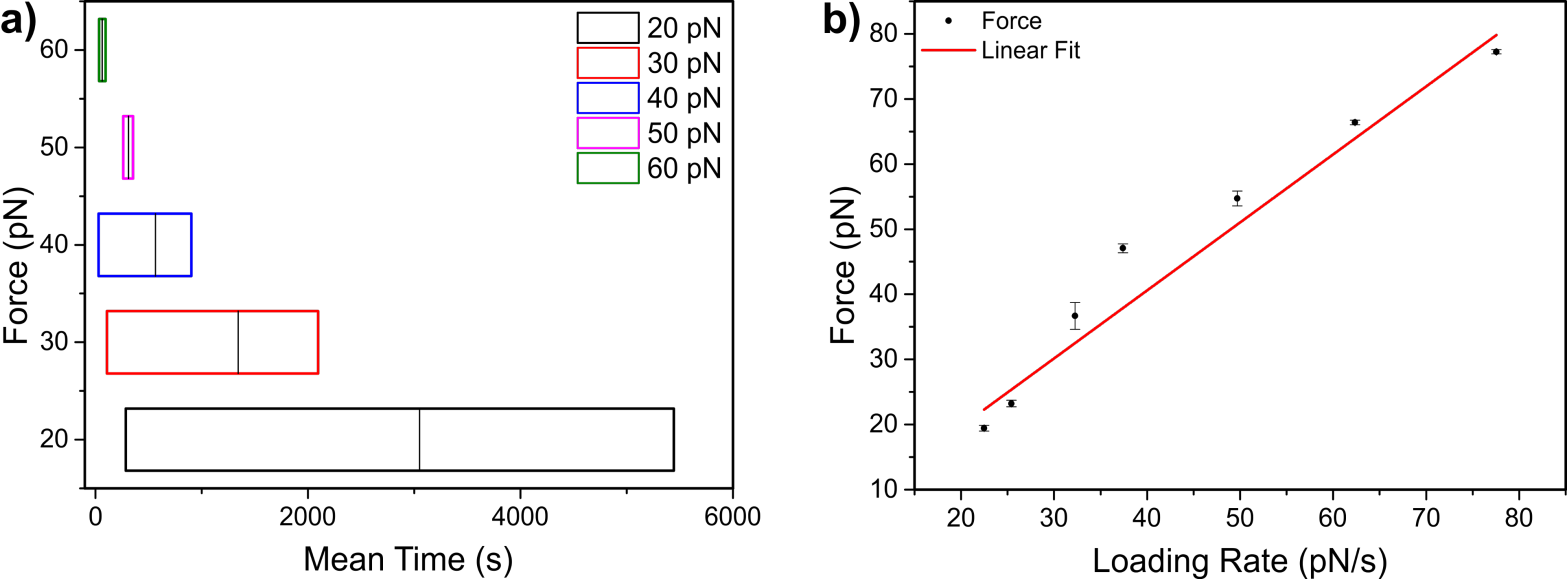
Studying the stability and force-induced disruption of streptavidin-labelled DNA handles in TICO buffer. a) Constant-force measurements were conducted to check the stability of the DIG::anti-DIG bond for a range of different applied tensions over time. The mean dissociation times to rupture individual PDHs are shown as vertical lines, the coloured boxes highlight the range of 25–75% of the fitted Gaussian distribution. b) Measured rupture forces plotted against loading rates. Laser tweezers analyses of streptavidin-labelled DNA handles identified rupture forces ranging from 25–110 pN during a constant elongation velocity of 100 nm/s, with correspondingly varying loading rates. The loading rate (pN/s) was highest shortly before the dsDNA underwent the overstretching transition. The individually measured rupture forces at a specific loading rate were averaged. Error bars represent the SD of the individual means.

We examined the dissociation time of PDH constructs with different lengths in TICO buffer with a constant bead-bead displacement by measuring the force as a function of time until the molecule ruptured (Figure 6a). With initial forces of 20 pN applied to 4056 bp streptavidin-labelled DHs we have checked the mean stability of DIG::anti-DIG bond in TICO buffer against biotinylated beads (Figure 1a). It can be clearly seen that at lower forces (20 pN) the mean stability and dissociation time of DIG::anti-DIG averaged around 50 min. Exposing the constructs to constant forces below 20 pN resulted in much longer stable connections. Increasing the constant applied forces on these particular constructs resulted in earlier rupturing of the tethers. Such long dissociation times will enable the use of these PDHs in constant force experiments in time critical motor protein studies. In Figure 6b, we show the rupture forces dependency of the streptavidin modified dsDNA handles on the loading rate [26–28] the results are comparable to a recent study of Sitters et al. [29].

### Protein–DNA handles for grasping single molecules

**Figure 7:**
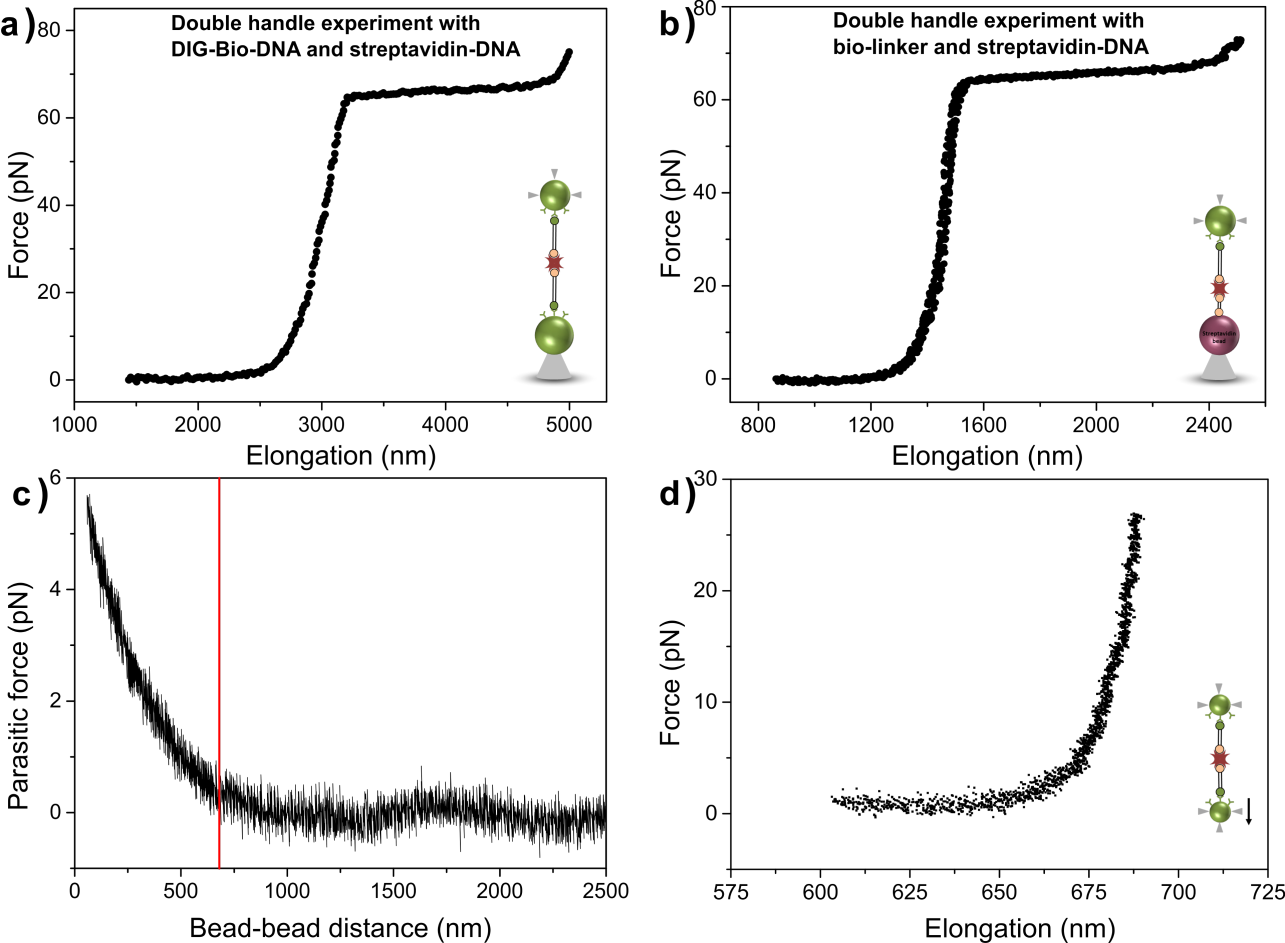
Characteristic force–extension curves of double-handle experiments. a) DIG-DNA-Bio and streptavidin-DNA, both tethered to anti-DIG beads (Figure 1d). With more than 1800 nm the plateau length was greater than 70% of the contour length of one DH, thus indicating a specific and stable connection between the two DNA molecules. b) Bio-linker modified streptavidin beads and streptavidin-DNA at anti-DIG bead with a plateau length of about 900 nm (Figure 1c). A stable tether was achieved, yielding a force–extension curve with a characteristic force plateau. c) Using dual-trap OT (Figure 1e) crosstalk is observed between two orthogonally polarized beams below a certain trap–trap distance (less than 700 nm separation betwenn the surfaces of the beads) due to polarization scrambling of the objectives resulting a parasitic signal. The red line indicates the optimum double DNA contour length of 680 nm (1000 bp each). d) Double-handle experiment using the dual-trap OT, where DHs and PDHs (1000 bp each) were tethered to small (0.84 μm) beads and tethered in situ. The assembly was stable over time and featured the characteristic double-handle contour length of 680 nm at a tension of approx. 10 pN.

As an application of our PDHs, we employed these constructs to grasp the biotin end of a DH as shown in Figure 1d,e. When connecting the two DHs the contour length of the newly formed double handle was expected to be doubled. This change in molecular length should also become apparent in the measured overstretch plateau length, which corresponds to approx. 70% of the contour length of a DNA molecule as previously reported [17,30–31]. Figure 7a shows a force–extension curve of a double handle formed by DHs and PDHs, each 4056 bp long (1379 nm each, Figure 1d). With more than 1800 nm the plateau length of this double DNA construct was twice the length of the characteristic value of 965 nm for one single handle. This double-plateau length indicates that the connection between the two molecules had successfully taken place in situ. Furthermore, we used PDHs with 4056 bp length to interconnect with short biotinylated dsDNA handles of 24 bp length, which were tethered to streptavidin spheres (Figure 1c). This experimental setup also yielded specific interactions with force–extension curves displaying characteristic force plateaus (Figure 7b). The successful tethering of short biotinylated DNA handles to streptavidin beads was confirmed in control experiments versus streptavidin spheres, which yielded up to 100% interactions. With the high resolution dual-trap OT (Figure 1e), using small beads (0.84 μm), optical crosstalk is observed between the two orthogonal polarized beams due to polarization scrambling within the objectives. The resulting parasitic signal, caused by interference between the scrambled portions of the two overlapping beams, can be seen in Figure 7c. Approximately 2% polarization scrambling was observed on both detectors [14] and the resulting differential crosstalk signal was obtained by increasing the distance between the two optically trapped beads. For nanomechanical experiments short molecular handles are preferred due to their increased mechanical stiffness, resulting in a favourable signal-to-noise ratio [14]. In order to achieve optimal positional and force resolution a balance had to be found between minimising the parasitic optical crosstalk signal in the dual-trap instrument and maximising the stiffness of the tethers. Double handle experiments with DNA handles of 1000 bp (contour lengths of more than 340 nm each), were the shortest possible constructs that still featured the least amount of parasitic cross-talk in measurements at forces above 10 pN using this particular dual-trap OT instrument. Measurements with the dual-trap tweezers using double handles (1000 bp each) yielded stable specific interactions and a representative force curve of such a double-handle construct is shown in Figure 7d. Thus, PDH constructs could be employed in such a dual-handle configuration to grasp doubly biotinylated molecules (or ensembles of molecules) in situ, for instance to tether a biotinylated ribosome exposing a stalled biotinylated nascent polypeptide chain. Using 0.84 μm beads and 1 kbp double handles, sub-nanometre position noise (FWHM) measurements were possible with the dual-trap tweezers for applied forces greater than 30 pN. Between 10 and 30 pN the measured position noise varied between 1 and 2 nm.

**Figure 8:**
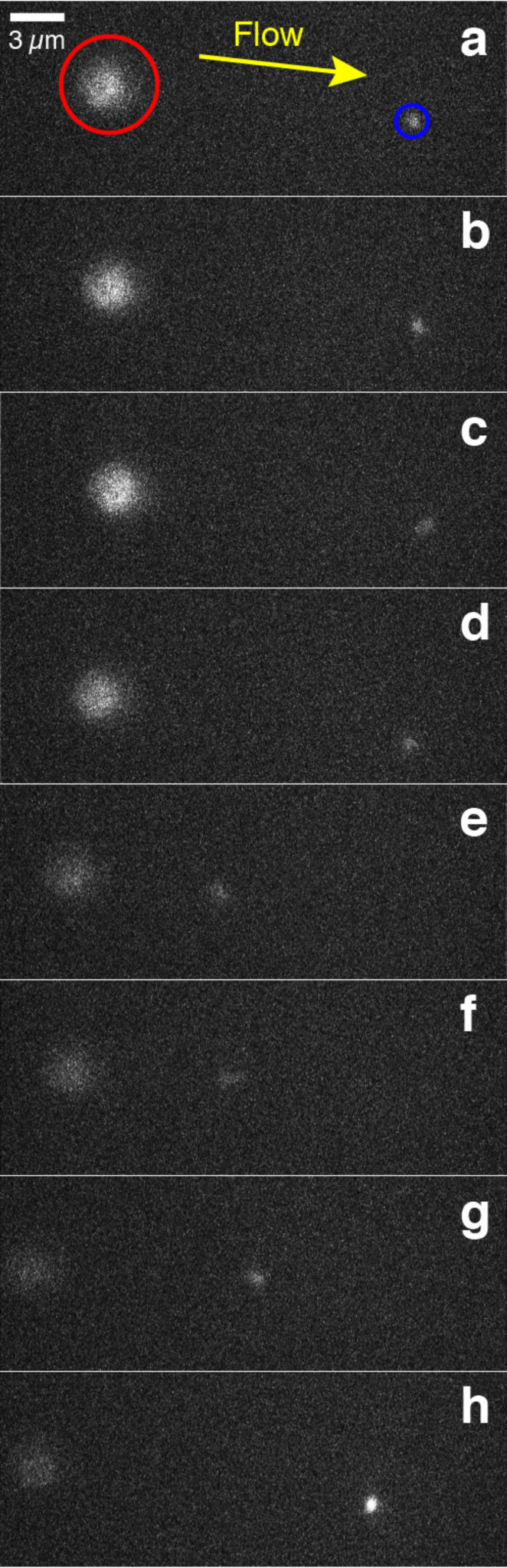
Fluorescence measurements of Qdot–streptavidin conjugates that were attached to freely accessible DIG -λ-dsDNA-Bio strands. a) The blue circle highlights the fluorescent emission of a quantum dot at 525 nm, and the red circle shows the auto-fluorescent bead on the pipette, the yellow arrow represents the direction of the flow. b–h) are the consecutive images obtained via EMCCD video recording. These images show one cycle of the relative position change of the tethered quantum dot, whilst bound to the biotinylated end of a single DIG-λ-DNA-Bio strand within the microfluidic chamber. It can be seen that the freely accessible Qdot changes its position according to the flow. a–c depict a constant fast fluid velocity. d–e show a sudden decrease and f–h a gradual increase in the flow rate within the microfluidic cell. Variations in the fluid flow speed change the hydrodynamic drag experienced by the long dsDNA molecule, altering its extension.

To study the efficiency of our protein-labelling procedure in more detail and to facilitate further single-molecule spectroscopy applications we have coupled streptavidin-modified quantum dots (Q-dot 525, Hayward CA) to the 3' end of DIG-λ-DNA-Bio constructs with 48502 bp length, which were tethered to anti-DIG beads held by suction with a pipette (Figure 1f). This experiment with λ-dsDNA was carried out as previously shown [13]. The Images in Figure 8 show that a streptavidin conjugate Q-dot is located at the free end of the biotin-labelled λ-dsDNA in the chamber and was excited using a 488 nm argon laser. Qdot emission at 525 nm was captured with an EMCCD camera and was recorded using IXON software (Andor, Belfast, NI). Multiple stretching and shortening cycles of the DNA strand due to variations in the fluid flow speeds were observed (10 cycles), as well as a variation in the emitted light intensity, which can be attributed either to moving of the Q-dot in and out of focus or the known blinking of fluorescing quantum dots [32] (Figure 8). This single molecule fluorescence experiment clearly visualizes the specific binding of a protein to the biotinylated end of the dsDNA. Since the dsDNA was not labelled with intercalating dyes in this fluorescence measurement only the Qdot streptavidin conjugate indicates the end of the modified DNA–protein hybrid.

## Conclusion

We present a simple and very reliable method to produce novel PDHs suitable as molecular handles for single molecule experiments. Gel electrophoresis confirmed the labelling procedure with either streptavidin or neutravidin. EMCCD imaging successfully shows that these protein-modified DNA ends are freely moving in solution by imaging the streptavidin conjugate Qdot linked to a long λ-dsDNA tethered to a streptavidin sphere in a liquid flow configuration. An optimal DNA-modification and purification yield was achieved with 100 proteins per handle. In optical force measurements with PDHs tethered to anti-DIG beads the highest number of single interactions without any clustering of the beads was obtained with a nominal ratio of 40 PDHs per anti-DIG bead. The protein-labelled DNA molecules repeatedly exhibited reliable mechanical characteristics in varying buffer conditions with rupture forces reaching maximum values of 120 pN. Furthermore, high dissociation times (longer than 50 min) were obtained when a constant force of 20 pN was applied to single protein–DNA molecule constructs. The length ranges of PDHs produced for single molecule experiments are defined by the optical tweezers set-up. Using dual-trap OT some optical (see Figure 1e) crosstalk is observed below a certain trap–trap distance. Here PDHs of a total length of approx. 700 nm are advised to minimize parasitic signals whilst maximizing the signal-to-noise ratio. The upper PDHs length limit is defined by the range of the steerable piezo-mirror (ca. 8 µm in the device used here). For tweezers systems utilizing a pipette and an optical trap (see Figure 1a–d) PDH molecules as short as 30 nm up to 30 µm can be investigated. Using these stable protein–DNA constructs of various lengths will greatly facilitate kinetic polypeptide elongation and unfolding/refolding experiments in future single molecular-motor studies involving the ribosome and will also be useful in the study of other biotinylated molecules and mechano-enzymes.
